# Elevated Plasma Chemokines for Eosinophils in Neuromyelitis Optica Spectrum Disorders during Remission

**DOI:** 10.3389/fneur.2018.00044

**Published:** 2018-02-12

**Authors:** Yanping Tong, Tao Yang, Jingwen Wang, Tianyou Zhao, Lei Wang, Yuezhi Kang, Cuicui Cheng, Yongping Fan

**Affiliations:** ^1^Department of Traditional Chinese Medicine, Beijing Tiantan Hospital, Capital Medical University, Beijing, China; ^2^TCM Brain Research Institution, Beijing Tiantan Hospital, Capital Medical University, Beijing, China; ^3^First Department of Neurology, Dongzhimen Hospital, Beijing University of Chinese Medicine, Beijing, China; ^4^School of Traditional Chinese Medicine, Capital Medical University, Beijing, China

**Keywords:** neuromyelitis optica spectrum disorders, CCL13, relapse, eotaxin, eosinophil, interleukin-1beta, tumor necrosis factor-alpha

## Abstract

**Background:**

A prominent pathological feature of neuromyelitis optica spectrum disorders (NMOSD) is markedly greater eosinophilic infiltration than that seen in other demyelinating diseases, like multiple sclerosis (MS). Eosinophils express the chemokine receptor CCR3, which is activated by eotaxins (CCL11/eotaxin-1, CCL24/eotaxin-2, CCL26/eotaxin-3) and CCL13 [monocyte chemoattractant protein (MCP)-4]. Moreover, CCL13 is part of the chemokine set that activates CCR2. The present study aimed to evaluate plasma levels of eotaxins (CCL11, CCL24, and CCL26) and MCPs (CCL13, CCL2, CCL8, and CCL7) in patients with NMOSD during remission.

**Methods:**

Healthy controls (HC; *n* = 30) and patients with MS (*n* = 47) and NMOSD (*n* = 58) in remission were consecutively enrolled in this study between January 2016 and August 2017. Plasma CCL11, CCL24, CCL26, CCL2, CCL8, CCL7, CCL13, tumor necrosis factor (TNF)-α, and interleukin (IL)-1β levels were detected using the human cytokine multiplex assay.

**Results:**

Plasma CCL13, CCL11, and CCL26 levels were all significantly higher in patients with NMOSD than in HC and patients with MS. No significant differences were found in the CCL13, CCL11, or CCL26 levels between patients with NMOSD receiving and not receiving immunosuppressive therapy. The plasma levels of TNF-α and IL-1β, which stimulate the above chemokines, were higher in patients with NMOSD than in HC. There was no difference in CCL24 levels among the three groups. In most cases, the CCL7 levels were below the threshold value of the human cytokine multiplex assay, which is in line with other studies. Adjusted multiple regression analyses showed a positive association of CCL13 levels with the number of relapses after controlling gender, age, body mass index, and disease duration in patients with NMOSD.

**Conclusion:**

The study indicates that in NMOSD, the overproduction of cytokines such as IL-1β and TNF-α during remission stimulates eosinophilic chemoattractants such as CCL13, CCL11, and CCL26, which in turn bind to their receptor (CCR3); this could lead to eosinophil hypersensitivity. These findings suggest that the elevated secretion of CCL13, CCL11, and CCL26 may be a critical step in eosinophil recruitment during NMOSD remission.

## Introduction

Neuromyelitis optica spectrum disorder (NMOSD), an idiopathic inflammatory demyelinating disorder of the central nervous system that involves both the optica nerves and the spinal cord, was considered a subtype of multiple sclerosis (MS) for many years until the discovery of its specific serum antibody biomarker—aquaporin (AQP) 4 IgG (1–3). Although B cells, AQP-4 antibody-dependent complement-dependent cellular cytotoxicity (CDCC), and antibody-mediated cellular cytotoxicity (ADCC) play decisive roles in the pathogenesis of NMOSD, eosinophils have been implicated as an important player in the pathological lesions following blood–brain barrier (BBB) injury (4–6). The basic histopathological features of NMOSD have been described as acute spinal cord lesions with diffuse swelling and softening over multiple segments involving the entire spinal cord or having continuous distribution (7). Autopsy examination has shown that a prominent feature of human NMOSD lesions is markedly greater eosinophilic infiltration than that seen with other demyelinating diseases like MS (8, 9). Eosinophils are also found in the cerebrospinal fluid (CSF) of patients with NMOSD, and demyelinating NMOSD lesions show marked eosinophil infiltration in patients with NMOSD and mouse models (4, 10–12). Lesion severity can increase because of transgenic hyper-eosinophilia and *vice versa* (4). An interesting finding is the presence of eosinophils in inflammatory demyelinating lesions in NMOSD but their absence in MS. Further, eosinophils have been found in all early active NMOSD lesions but not in cases of acute disseminated encephalomyelitis or acute spinal cord infarction (8). Other studies have proved that the lesion infiltrates are associated with C–C motif chemokine receptor (CCR)-3 expression and stain positively for major basic protein (MBP) in eosinophils, suggesting basic protein release from granules in patients with NMOSD.

CCR3 is highly expressed on the surface of eosinophils. Interactions of CCR3 with a variety of chemokines including CCL11 (eotaxin-1), CCL24 (eotaxin-2), CCL26 (eotaxin-3), CCL13 (monocyte chemoattractant protein 4, MCP-4), CCL8 (MCP-2), and CCL7 (MCP-3) play an important role in the migration, accumulation, and activation of eosinophils (13–15). The “eotaxins,” CCL11, CCL24, and CCL26, represent selective chemoattractants for eosinophils (14). Together with CCL11, CCL13 is one of the most important eosinophilic chemoattractants (16). This protein homeostatically recruits eosinophils to organs in disease states. In humans, four MCPs have been identified: MCP-1 (CCL2), MCP-2 (CCL8), MCP-3 (CCL7), and MCP-4 (CCL13) (17). Unlike other MCPs, CCL13 binds not only with CCR1 and CCR2 but also with CCR3, to act as a chemoattractant for eosinophils (18, 19). Several lines of evidence have shown that CCL13 plays a pivotal role in inflammatory cell recruitment in allergic and autoimmune disorders such as rheumatoid arthritis, asthma, parasitic infection, and atopic dermatitis, and it also works in overweight subjects (20–24).

The present study aimed to investigate the factors that induce the effects of eosinophils in NMOSD during remission. Thirty healthy controls (HC), 47 patients with MS, and 58 patients with NMOSD were enrolled to screen cytokines and chemokines. The study showed that plasma levels of tumor necrosis factor-alpha (TNF-α), interleukin (IL)-1β, CCL13, CCL11, and CCL26 were increased in patients with NMOSD. Additional analysis led to the finding that CCL13 levels were strongly correlated with recurrence times, and we believe that it can be considered one of the most valuable prognostic factors in NMOSD.

## Subjects and Methods

### Subjects

Ethics approval for this study was obtained from the ethics committee of the Beijing Tiantan Hospital Affiliated to the Capital Medical University in China (No. KY2015-003-02). Prior to participation, all patients and HC provided written informed consent.

Patients with NMOSD and MS were recruited from the Beijing Tiantan Hospital between January 2016 and August 2017. The diagnosis of these conditions was confirmed according to the 2015 revised international criteria (25) and the 2010 McDonald’s diagnostic criteria (26), respectively. All patients were in remission (had remained relapse-free for over a 1-month period) and were not coexisting any other autoimmune comorbidities at the time of blood collection. Kutzke Expand Disability Status Scale (EDSS) scores were determined from an MS cohort study. Age- and sex-matched volunteers without immune disorders were recruited as HC. Infections were ruled out on the basis of complete blood count testing in all subjects.

### Assay for Plasma Chemokines and Cytokine Levels

Peripheral blood was obtained from each subject. To exclude the effect of different time points and other factors on the level of chemokines and cytokines, all blood samples were collected at 9:00 a.m. After collection into a 4-ml disposable BD Vacutainer^®^ containing ethylene diamine tetraacetic acid, plasma samples were separated at 2,000 × *g* for 10 min within 3 h and stored in aliquots at −80°C until further analysis. All testing was performed in a blinded manner to the diagnosis or clinical presentations.

Plasma CCL11, CCL24, and CCL26; CCL2, CCL8, CCL7, and CCL13; and TNF-α and IL-1β levels were assayed using MILLIPLEX^®^ map human High Sensitivity Cytokine/Chemokine Panels (Cat. Nos. HCYTOMAG-60K and HCYP2MAG-62K) (Merck KGaA, Darmstadt, Germany) according to the manufacturer’s instructions.

### Statistical Analysis

Statistical analysis was conducted using SPSS 22.0 (International Business Machines Corporation, Chicago, IL, USA). After the test of normality, data with non-Gaussian distributions were analyzed using the Mann–Whitney *U*-test for two groups and the Kruskal–Wallis *H*-test for multiple groups by using Dunn’s *post hoc* analysis. Normally distributed data were processed using Student’s *t* test or analysis of variance. CCL13, CCL11, and CCL26 values of NMOSD patients were used as independent variables in multiple linear regression analysis, with relapse times, annual relapse rate (ARR), and EDSS scores as continuous outcome measure. Gender, age, body mass index (BMI), and duration of NMOSD were included to determine the variables independently associated with these outcomes. Correlations between TNF-α, IL-1β, and CCL13, CCL11, and CCL26 were assessed using nonparametric Spearman’s rank test. A *P*-value of <0.05 was considered to be statistically significant.

## Results

### Clinical Demographics

We identified 58 patients with NMOSD, 47 patients with MS, and 30 HC, and their demographic and clinical characteristics are described in Table 1. The disease duration and duration to the last relapse in the patients with NMOSD were not significantly longer than that in patients with MS. A significant difference in EDSS scores was found between the NMOSD and MS groups (*P* < 0.01), which was consistent with our previous findings (27).

**Table 1:** Patient demographic and clinical characteristics.

Index	HC	MS	NMOSD
No. of patients	30	47	58
Gender (female/male)	22/8	34/13	54/4
Age (years, mean ± SE)	33.90 ± 1.64	34.96 ± 1.59	39.53 ± 1.57
Age at onset (years, median, and range)	–	30, 6–60	35, 14–62
BMI (kg/m^2^, mean ± SE)	21.58 ± 0.44	22.36 ± 0.43	22.80 ± 0.42
Duration of disease (months, mean, and range)	–	40.36, 1–143	56.11, 2–260
Duration to the last relapse (months, mean, and range)	–	5.79, 1–57	5.91, 1–33
No. of relapses (mean ± SE)	–	2.85 ± 0.26	3.35 ± 0.23
ARR (mean ± SE)	–	2.17 ± 0.37	1.62 ± 0.20
EDSS	–	2.64 ± 0.20	3.54 ± 0.21**

*HC, healthy controls; MS, multiple sclerosis; NMOSD, neuromyelitis optica spectrum disorders; No., number, BMI, body mass index; EDSS, expanded disability status scale; ARR, annual relapse rate*.

**P < 0.01.

### Plasma MCPs (CCL2, CCL8, CCL7, and CCL13) and Eotaxins (CCL11, CCL24, and CCL26) Levels in NMOSD

**Figure 1:**
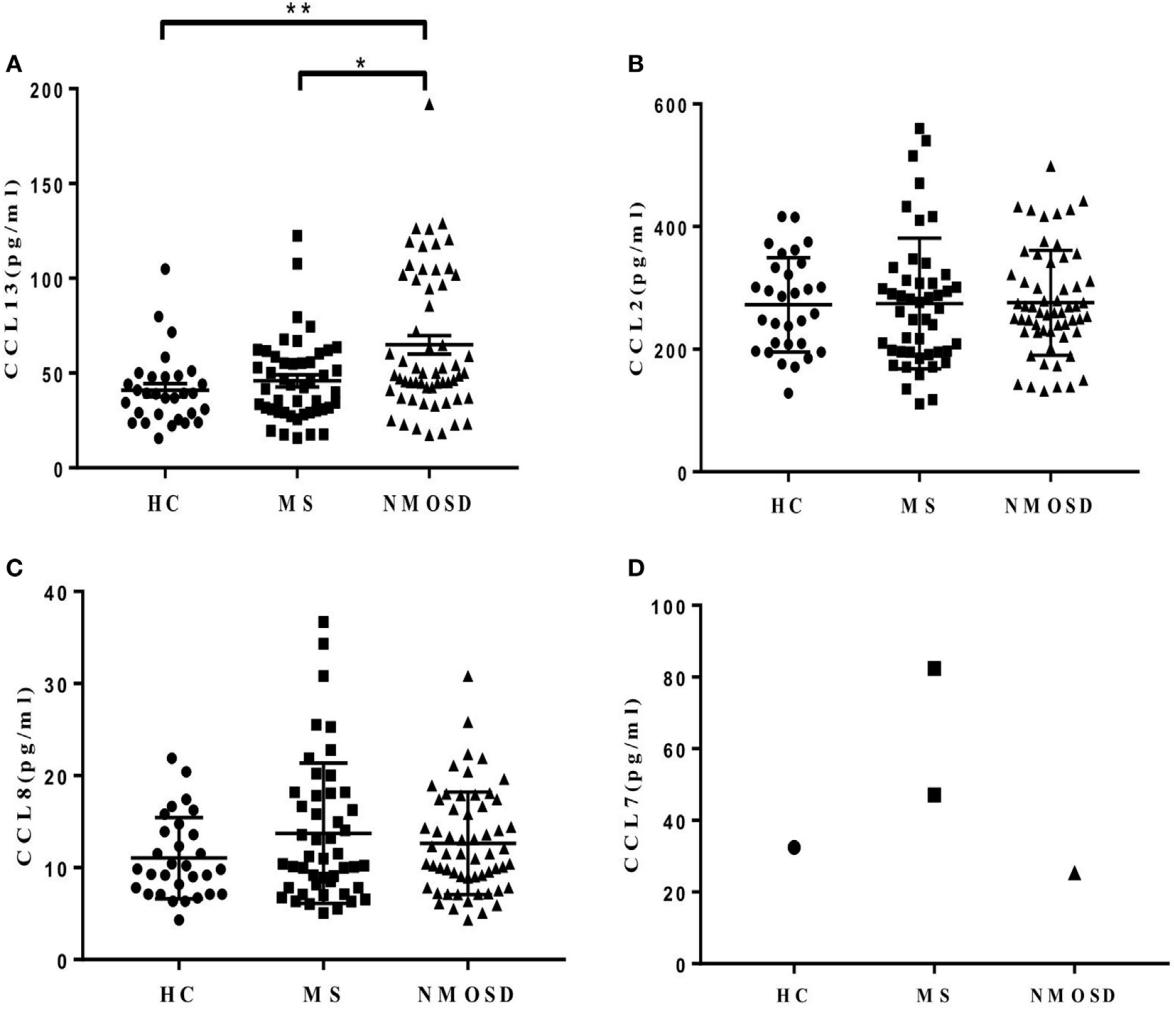
Plasma levels of monocyte chemoattractant proteins (CCL13, CCL2, CCL8, and CCL7). **(A)** Plasma CCL13 levels in healthy control (HC), multiple sclerosis (MS) patients, and neuromyelitis optica spectrum disorder (NMOSD) patients (mean ± SE). **(B,C)** No significant differences of CCL2 and CCL8 levels were found among plasma in HC, MS patients, and NMOSD patients (mean ± SE) (*P* = 0.89 and 0.39, respectively). **(D)** Plasma CCL7 levels in almost all subjects were below measurable levels. Kruskal–Wallis *H*-test and Dunn’s *post hoc* analysis were used. *P < 0.05, **P < 0.01.

Plasma CCL13 levels were significantly higher in patients with NMOSD than in HC and patients with MS. The levels in patients with MS were not significantly higher than in HC (Figure 1A). We also examined the levels of other MCPs in all subjects. Unlike CCL13, no significant differences were found in the levels of CCL2 and CCL8 among patients with NMOSD, patients with MS, and HC (Figures 1B,C). Further, the CCL7 levels in almost all subjects were below measurable levels (Figure 1D).

**Figure 2:**
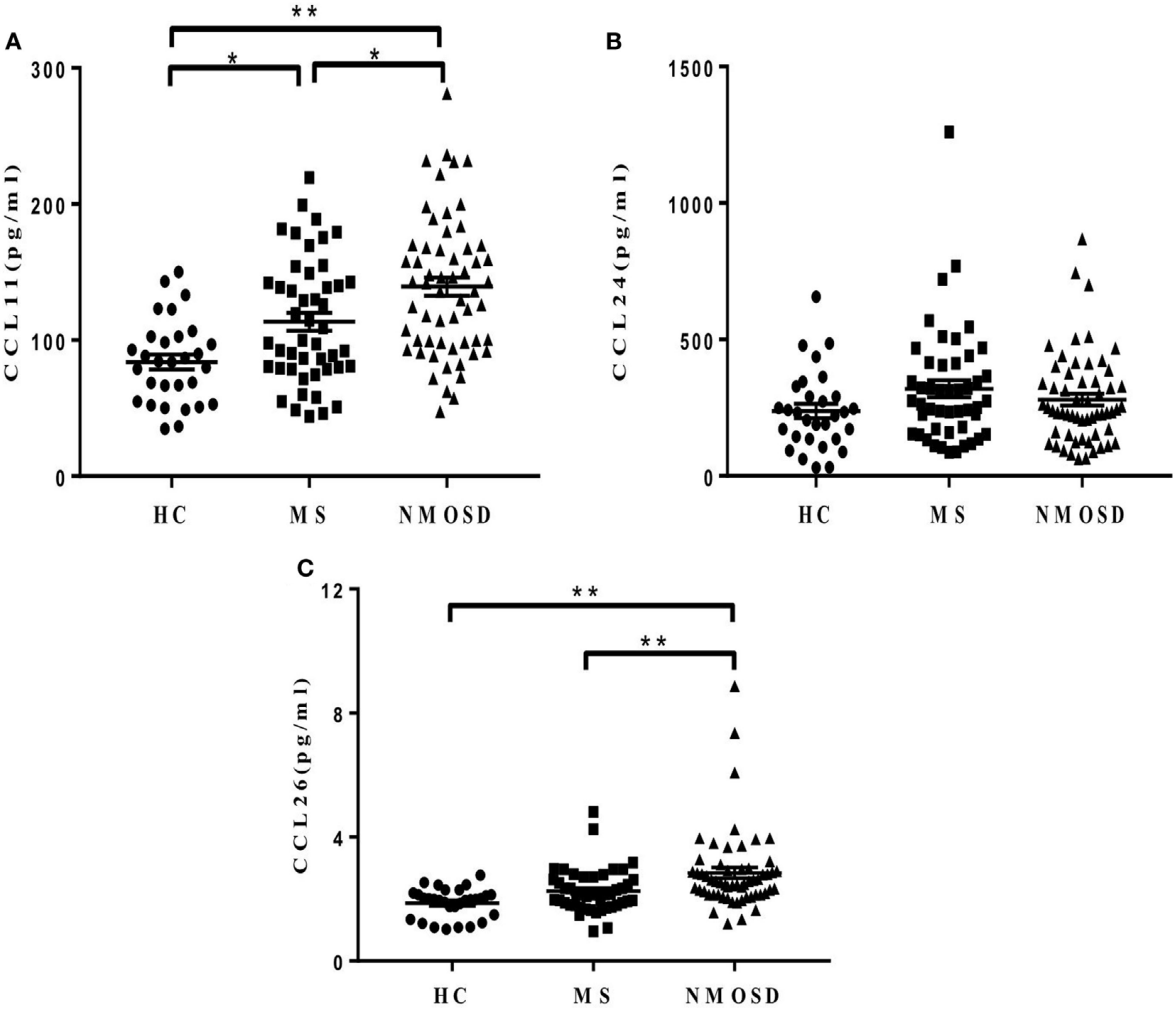
Plasma levels of eotaxins (CCL11, CCL24, and CCL26). **(A,C)** Different plasma levels of CCL11 and CCL26 levels in healthy control (HC), multiple sclerosis (MS) patients, and neuromyelitis optica spectrum disorder (NMOSD) patients (mean ± SE), and **(B)** no significant differences of CCL24 levels were found among the three groups (mean ± SE) (*P* = 0.18). Kruskal–Wallis *H*-test and Dunn’s *post hoc* analysis were used. *P < 0.05, **P < 0.01.

Plasma CCL11 levels in patients with NMOSD during remission were significantly higher than those in patients with MS and HC, and CCL26 levels showed similar findings. Patients with MS had higher CCL11 levels than the HCs did, but no significant differences were found in CCL26 levels between them (Figures 2A,C). No significant differences were found in plasma CCL24 levels among the three groups (Figure 2B). Similar results were found when removing the NMOSD outliers in CCL13, CCL11, and CCL26 concentrations and data were shown in Figure S3 in Supplementary Material.

### Effect of Immunosuppressive Therapy on Plasma Levels of CCL13, CCL11, and CCL26 in NMOSD

Eighty-three percent of the recruited patients with NMOSD were undergoing immunosuppressive therapy [*n* = 48; 27 with oral glucocorticoids and 21 with oral treatment with mycophenolate mofetil, azathioprine, tocilizumab, or cyclosporine A (14 patients received one of these drugs in combination with glucocorticoids)]. Therefore, it was necessary to study whether immunosuppressive therapy affected plasma CCL13, CCL11, and CCL26 levels. The plasma CCL13, CCL11, and CCL26 levels in patients with and without immunosuppressive therapy were both significantly higher than those in the HC, and the levels in patients undergoing immunosuppressive therapy were not significantly lower than those in the other 10 patients (Figure S1 in Supplementary Material). These findings indicated that immunosuppressive therapy had little effect on the three eosinophilic chemoattractant levels.

### Regression Analysis of CCL13, CCL11, and CCL26 with Relapse Times, ARR, and EDSS Scores in NMOSD

In NMOSD patients, multiple regression analysis of CCL13, CCL11, and CCL26 with relapse times, ARR, and EDSS scores showed that only CCL13 was significantly related to relapse times (*R^2^* = 0.553, *P* = 0.000) after controlling gender, age, BMI, and disease duration (Table 2). Age and disease duration were identified as independent factors associated with relapse times (*P* = 0.029 and 0.024, respectively). Gender and BMI showed no independent and significant regression coefficient with relapse times (*P* = 0.158 and 0.355, respectively). No associations of CCL11, CCL13, and CCL26 with ARR or EDSS scores were found. These data suggested that plasma CCL13 levels may have a prognostic value in NMOSD. Further studies with a larger number of subjects would provide adequate power to detect the differential correlation pattern between CCL13 levels and the clinical characteristics of NMOSD. Similar results were found after removing the NMOSD outliers in CCL13, CCL11, and CCL26 concentrations (Table 1 in Supplementary Material).

**Table 2:** Adjusted regression coefficients (β) of CCL13, CCL11, and CCL26 levels with relapse times, ARR, and EDSS scores as outcomes in neuromyelitis optica spectrum disorder patients.

Chemokines	Outcomes	Adjusted β	*R*^2^	*P*-values	Adjusted for
CCL13	Relapse times	0.571	0.553	0.000	Age, disease duration
	ARR	0.002	0.331	0.985	Disease duration
	EDSS	0.007	0.179	0.959	Age

CCL11	Relapse times	0.015	0.274	0.903	Age, disease duration
	ARR	−0.128	0.346	0.281	Disease duration
	EDSS	0.051	0.181	0.700	Age

CCL26	Relapse times	−0.032	0.274	0.803	Age, disease duration
	ARR	0.017	0.331	0.891	Disease duration
	EDSS	−0.031	0.180	0.820	Age

*ARR, annual relapse rate; EDSS, expanded disability status scale*.

### Plasma TNF-α and IL-1β Levels and Their Correlation with CCL13, CCL11, and CCL26 Levels

**Figure 3:**
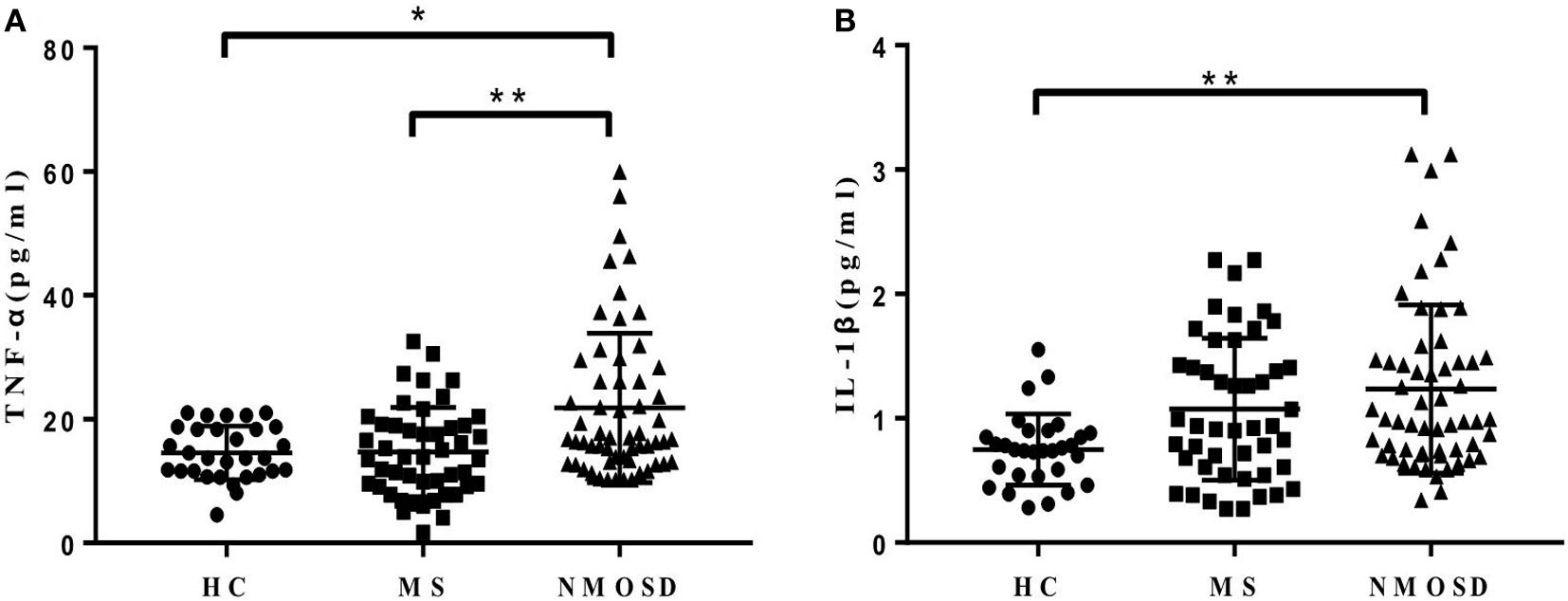
**(A,B)** Plasma levels of tumor necrosis factor-alpha (TNF-α) and interleukin-1β (IL-lβ) in healthy control (HC), multiple sclerosis (MS) patients, and neuromyelitis optica spectrum disorder (NMOSD) patients. Kruskal–Wallis *H*-test and Dunn’s *post hoc* analysis were used. *P < 0.05, **P < 0.01.

In order to explore the mechanisms underlying the increased CCL13, CCL11, and CCL26 levels in NMOSD, we examined the levels of plasma TNF-α and IL-1β, which have been proposed to stimulate their production. The data showed that both plasma TNF-α and IL-1β levels in patients with NMOSD were significantly higher than those in HC. Further, the TNF-α levels were lower in patients with MS than in those with NMOSD (Figures 3A,B). Plasma TNF-α and IL-1β levels were positively correlated with plasma CCL13, and TNF-α levels were also positively correlated with plasma CCL11 levels (Figure S2 in Supplementary Material). No correlation was found between IL-1β and CCL11 (*P* = 0.299), and neither TNF-α nor IL-1β levels showed correlations with CCL26 (*P* = 0.108 and 0.297, respectively).

## Discussion

Recently available data suggest that low-grade inflammation plays a pivotal role in the development of NMOSD, and low-grade inflammation is frequently observed in patients with NMOSD. As a source of versatile proinflammatory mediators, eosinophils have the unique characteristic-specific granules, which contain four major granule proteins (MBP, eosinophil cationic protein, eosinophil peroxidase, and eosinophil neurotoxin) and numerous cytokines, chemokines, and growth factors (28). These granule releases are the involved infiltrates of eosinophilic ADCC and CDCC response. Histopathologic studies had demonstrated that marked perivascular and meningeal eosinophil infiltrations were showed in active NMOSD lesions and were associated with CCR3 expression (8). CCR3 is a member of the seven transmembrane domain G protein-coupled receptor family, and its major ligands—CCL13 and eotaxins (CCL11, CCL24, and CCL26)—are involved in eosinophil chemoattraction (29).

Similar to other MCPs, CCL13 binds to CCR1 and CCR2 and acts as a chemoattractant for monocytes, T cells, and dendritic cells (DCs), and it is the only MCP that binds to CCR3. Together with CCL11, CCL13 is one of the most important chemoattractants for eosinophils (16). Considering the roles that eosinophils play in NMOSD pathogenesis, CCL13 may act as a distinctive chemoattractant in NMOSD. Compared to other MCPs, CCL13 plays an interesting role in DC migration into inflamed lesions, because monocytes and blood DC precursors such as CD34+-derived DCs and CD11c + DCs respond first to CCR2 ligands and CCL13 in epithelial cells present in contracted blood vessels, and these cells could then be recruited into inflamed tissue by a different gradient (30, 31). In addition, one subtype of Th17 cells that could induce the production of the AQP-4 antibody is distinguishable from its expression of CCR2 + CCR5− and RORγt, and ligands of CCR2 like CCL13 may be important regulators in the recruitment of Th17 cells in NMOSD pathogenesis (32). In the present study, we demonstrated for the first time that the level of the MCP-4 protein is elevated in the sera of patients with NMOSD. This elevation may in turn activate DCs and Th17 cells, especially eosinophils, which could worsen inflammation and lead to recurrence (33). In addition, the association of CCL13 levels with the number of relapses was analyzed, and the results showed a significant positive relationship between them. This finding indicated that CCL13 may play an extensive pathological role during remission in NMOSD. On the basis of these findings, we suggest that blocking the actions of CCL13 might serve as a novel strategy for the generation of agents with anti-inflammatory activity during remission in patients with NMOSD.

Other MCPs that bind with CCR1 and CCR2 to achieve signal cascade were also assayed in the present study. The results showed no significant changes in CCL2 and CCL7 levels during remission in patients with NMOSD. Further, the CCL7 levels were too low to measure in most patients, which is in line with the findings of most studies (34).

Several studies identified higher levels of eotaxins in the CSF and serum of patients with NMOSD than those in MS patients during acute attacks and concluded that this may be responsible for eosinophil migration across the BBB (10, 35); however, few studies focus on eotaxins levels during remission. The present study showed that plasma CCL11 and CCL26 were upregulated in patients with NMOSD during remission, implying that CCL11 and CCL26 may persistently participate in eosinophil activation, recruitment, and amplification in the pathogenesis of NMOSD after acute attacks. However, CCL24, which is also a strong chemoattractant for eosinophils, was not upregulated during the remission stage. Furthermore, no correlation was found between CCL11 levels, as well as CCL26, and clinical characteristics of patients with NMOSD.

Moreover, TNF-α and IL-1β were examined in this study for their ability to stimulate the release of multiple cytokines including CCL13 and eotaxins through the extracellular signal-regulated kinase cascade (18, 36–40). The results showed that the levels of both these inflammatory markers were elevated in the plasma of patients with NMOSD during remission. This indicated that TNF-α and IL-1β may be involved in the pathogenesis NMOSD mediated by CCL13, CCL11, and CCL26.

Collectively, the findings of the present study indicated that CCL13, CCL11, and CCL26 were upregulated by TNF-α and IL-1β, and this in turn led to the activation of eosinophils. This may have worsened the inflammation, leading to relapses in patients with NMOSD during remission.

## Ethics Statement

Ethics approval for this study was obtained from the ethics committee of the Beijing Tiantan Hospital Affiliated to the Capital Medical University in China (No. KY2015-003-02). Prior to participation, all patients and healthy controls provided written informed consent.

## Author Contributions

YT, TY, and YF conceived and designed the study. YT, TY, JW, and TZ performed the MILLIPLEX^®^ map human High Sensitivity Cytokine/Chemokine Panels tests, and participated in evaluating the EDSS scores of patients and collecting blood samples. LW, YK, and CC performed the statistical analysis. YF and LW revised the manuscript. All authors reviewed the final manuscript.

## Conflict of Interest Statement

The authors declare that the research was conducted in the absence of any commercial or financial relationships that could be construed as a potential conflict of interest.

## References

[1] Takahashi T Fujihara K Nakashima I Misu T Miyazawa I Nakamura M Anti-aquaporin-4 antibody is involved in the pathogenesis of NMO: a study on antibody titre. Brain (2007) 130:1235–43. 10.1093/brain/awm062 17449477

[2] Saikali P Cayrol R Vincent T. Anti-aquaporin-4 auto-antibodies orchestrate the pathogenesis in neuromyelitis optica. Autoimmun Rev (2009) 9:132–5. 10.1016/j.autrev.2009.04.004 19389490

[3] Tradtrantip L Zhang H Saadoun S Phuan PW Lam C Papadopoulos MC Anti-aquaporin-4 monoclonal antibody blocker therapy for neuromyelitis optica. Ann Neurol (2012) 71:314–22. 10.1002/ana.22657 22271321 PMC3314396

[4] Zhang H Verkman AS. Eosinophil pathogenicity mechanisms and therapeutics in neuromyelitis optica. J Clin Invest (2013) 123:2306–16. 10.1172/JCI67554 23563310 PMC3635742

[5] Tradtrantip L Ratelade J Zhang H Verkman AS. Enzymatic deglycosylation converts pathogenic neuromyelitis optica anti-aquaporin-4 immunoglobulin G into therapeutic antibody. Ann Neurol (2013) 73:77–85. 10.1002/ana.23741 23055279 PMC3567850

[6] Phuan PW Anderson MO Tradtrantip L Zhang H Tan J Lam C A small-molecule screen yields idiotype-specific blockers of neuromyelitis optica immunoglobulin G binding to aquaporin-4. J Biol Chem (2012) 287:36837–44. 10.1074/jbc.M112.408716 22989877 PMC3481286

[7] Hinson SR Roemer SF Lucchinetti CF Fryer JP Kryzer TJ Chamberlain JL Aquaporin-4-binding autoantibodies in patients with neuromyelitis optica impair glutamate transport by down-regulating EAAT2. J Exp Med (2008) 205:2473–81. 10.1084/jem.20081241 18838545 PMC2571922

[8] Lucchinetti CF Mandler RN McGavern D Bruck W Gleich G Ransohoff RM A role for humoral mechanisms in the pathogenesis of Devic’s neuromyelitis optica. Brain (2002) 125:1450–61. 10.1093/brain/awf151 12076996 PMC5444467

[9] Michael BD Elsone L Griffiths MJ Faragher B Borrow R Solomon T Post-acute serum eosinophil and neutrophil-associated cytokine/chemokine profile can distinguish between patients with neuromyelitis optica and multiple sclerosis; and identifies potential pathophysiological mechanisms – a pilot study. Cytokine (2013) 64:90–6. 10.1016/j.cyto.2013.07.019 23941778

[10] Correale J Fiol M. Activation of humoral immunity and eosinophils in neuromyelitis optica. Neurology (2004) 63:2363–70. 10.1212/01.WNL.0000148481.80152.BF 15623701

[11] Saadoun S Waters P Leite MI Bennett JL Vincent A Papadopoulos MC. Neuromyelitis optica IgG causes placental inflammation and fetal death. J Immunol (2013) 191:2999–3005. 10.4049/jimmunol.1301483 23935196 PMC4161708

[12] Matsushita T Tateishi T Isobe N Yonekawa T Yamasaki R Matsuse D Characteristic cerebrospinal fluid cytokine/chemokine profiles in neuromyelitis optica, relapsing remitting or primary progressive multiple sclerosis. PLoS One (2013) 8:e61835. 10.1371/journal.pone.0061835 23637915 PMC3630114

[13] Pease JE. Targeting chemokine receptors in allergic disease. Biochem J (2011) 434:11–24. 10.1042/BJ20101132 21269275

[14] Bachelerie F Ben-Baruch A Burkhardt AM Combadiere C Farber JM Graham GJ International union of basic and clinical pharmacology. LXXXIX. Update on the extended family of chemokine receptors and introducing a new nomenclature for atypical chemokine receptors. Pharmacol Rev (2014) 66:1–79. 10.1124/pr.113.007724 24218476 PMC3880466

[15] Zlotnik A Yoshie O Chemokines: a new classification system and their role in immunity. Immunity (2000) 12:121–7. 10.1016/S1074-7613(00)80165-X 10714678

[16] Mendez-Enriquez E García-Zepeda EA. The multiple faces of CCL13 in immunity and inflammation. Inflammopharmacology (2013) 21:397–406. 10.1007/s10787-013-0177-5 23846739

[17] Luster AD Rothenberg ME. Role of the monocyte chemoattractant protein and eotaxin subfamily of chemokines in allergic inflammation. J Leukoc Biol (1997) 62:620–33. 10.1002/jlb.62.5.620 9365117

[18] Garcia-Zepeda EA Combadiere C Rothenberg ME Sarafi MN Lavigne F Hamid Q Human monocyte chemoattractant protein (MCP)-4 is a novel CC chemokine with activities on monocytes, eosinophils, and basophils induced in allergic and nonallergic inflammation that signals through the CC chemokine receptors (CCR)-2 and -3. J Immunol (1996) 157:5613–26. 8955214

[19] Godiska R Chantry D Raport CJ Schweickart VL Trong HL Gray PW. Monocyte chemotactic protein-4: tissue-specific expression and signaling through CC chemokine receptor-2. J Leukoc Biol (1997) 61:353–60. 10.1002/jlb.61.3.353 9060459

[20] Hashimoto I Wada J Hida A Baba M Miyatake N Eguchi J Elevated serum monocyte chemoattractant protein-4 and chronic inflammation in overweight subjects. Obesity (Silver Spring) (2006) 14:799–811. 10.1038/oby.2006.93 16855189

[21] Lisi S Sisto M Lofrumento DD D’Amore M Sjögren’s syndrome autoantibodies provoke changes in gene expression profiles of inflammatory cytokines triggering a pathway involving TACE/NF-κB. Lab Invest (2012) 92:615–24. 10.1038/labinvest.2011.190 22157716

[22] Stellato C Collins P Ponath PD Soler D Newman W La Rosa G Production of the novel C-C chemokine MCP-4 by airway cells and comparison of its biological activity to other C-C chemokines. J Clin Invest (1997) 99:926–36. 10.1172/JCI119257 9062350 PMC507900

[23] Lechner CJ Komander K Hegewald J Huang X Gantin RG Soboslay PT Cytokine and chemokine responses to helminth and protozoan parasites and to fungus and mite allergens in neonates, children, adults, and the elderly. Immun Ageing (2013) 10:29. 10.1186/1742-4933-10-29 23855879 PMC3720251

[24] Iwamoto T Okamoto H Kobayashi S Ikari K Toyama Y Tomatsu T A role of monocyte chemoattractant protein-4 (MCP-4)/CCL13 from chondrocytes in rheumatoid arthritis. FEBS J (2007) 274:4904–12. 10.1111/j.1742-4658.2007.06013.x 17824960

[25] Wingerchuk DM Banwell B Bennett JL Cabre P Carroll W Chitnis T International consensus diagnostic criteria for neuromyelitis optica spectrum disorders. Neurology (2015) 85:177–89. 10.1212/WNL.0000000000001729 26092914 PMC4515040

[26] Polman CH Reingold SC Banwell B Clanet M Cohen JA Filippi M Diagnostic criteria for multiple sclerosis: 2010 revisions to the McDonald criteria. Ann Neurol (2011) 69:292–302. 10.1002/ana.22366 21387374 PMC3084507

[27] Yang T Wang S Zheng Q Wang L Li Q Wei M Increased plasma levels of epithelial neutrophil-activating peptide 78/CXCL5 during the remission of Neuromyelitis optica. BMC Neurol (2016) 16:96. 10.1186/s12883-016-0622-3 27401736 PMC4940958

[28] Rosenberg HF Dyer KD Foster PS. Eosinophils: changing perspectives in health and disease. Nat Rev Immunol (2013) 13:9–22. 10.1038/nri3341 23154224 PMC4357492

[29] Diny NL Rose NR Čiháková D. Eosinophils in autoimmune diseases. Front Immunol (2017) 8:484. 10.3389/fimmu.2017.00484 28496445 PMC5406413

[30] Osterholzer JJ Ames T Polak T Sonstein J Moore BB Chensue SW CCR2 and CCR6, but not endothelial selectins, mediate the accumulation of immature dendritic cells within the lungs of mice in response to particulate antigen. J Immunol (2005) 175:874–83. 10.4049/jimmunol.175.2.874 16002685 PMC2396199

[31] Vanbervliet B Homey B Durand I Massacrier C Aït-Yahia S de Bouteiller O Sequential involvement of CCR2 and CCR6 ligands for immature dendritic cell recruitment: possible role at inflamed epithelial surfaces. Eur J Immunol (2002) 32:231–42. 10.1002/1521-4141(200201)32:1<231::AID-IMMU231>3.0.CO;2-8 11782014

[32] Aranami T Yamamura T. Th17 cells and autoimmune encephalomyelitis (EAE/MS). Allergol Int (2008) 57:115–20. 10.2332/allergolint.R-07-159 18427164

[33] Saadoun S Bridges LR Verkman AS Papadopoulos MC. Paucity of natural killer and cytotoxic T cells in human neuromyelitis optica lesions. Neuroreport (2012) 23:1044–7. 10.1097/WNR.0b013e32835ab480 23108041 PMC3590012

[34] Lechner CJ Gantin RG Seeger T Sarnecka A Portillo J Schulz-Key H Chemokines and cytokines in patients with an occult *Onchocerca volvulus* infection. Microbes Infect (2012) 14:438–46. 10.1016/j.micinf.2011.12.002 22202179

[35] Correale J Fiol M. Chitinase effects on immune cell response in neuromyelitis optica and multiple sclerosis. Mult Scler (2011) 17:521–31. 10.1177/1352458510392619 21159721

[36] Boehme SA Sullivan SK Crowe PD Santos M Conlon PJ Sriramarao P Activation of mitogen-activated protein kinase regulates eotaxin-induced eosinophil migration. J Immunol (1999) 163:1611–8. 10415066

[37] Matsukura S Stellato C Plitt JR Bickel C Miura K Georas SN Activation of eotaxin gene transcription by NF-kappa B and STAT6 in human airway epithelial cells. J Immunol (1999) 163:6876–83. 10586089

[38] Wong CK Zhang JP Ip WK Lam CW. Activation of p38 mitogen-activated protein kinase and nuclear factor-kappaB in tumour necrosis factor-induced eotaxin release of human eosinophils. Clin Exp Immunol (2002) 128:483–9. 10.1046/j.1365-2249.2002.01880.x 12067303 PMC1906250

[39] Chakravorty SJ Howie AJ Girdlestone J Gentle D Savage CO. Potential role for monocyte chemotactic protein-4 (MCP-4) in monocyte/macrophage recruitment in acute renal inflammation. J Pathol (2001) 194:239–46. 10.1002/path.877 11400154

[40] Lampinen M Carlson M Håkansson LD Venge P. Cytokine-regulated accumulation of eosinophils in inflammatory disease. Allergy (2004) 59:793–805. 10.1111/j.1398-9995.2004.00469.x 15230810

