# Supplementary material for: Elevated Plasma Chemokines for Eosinophils in Neuromyelitis Optica Spectrum Disorders during Remission
Source: Front Neurol. 2018 Feb 12;9:44. doi: 10.3389/fneur.2018.00044 (PMC5819570; doi:10.3389/fneur.2018.00044)
Supplement: Supplementary file 1 [file table_1.PDF]

Supplemental Table 1. Adjusted regression coefficients (β) of CCL13, CCL11 and CCL26 levels with relapse times, ARR and EDSS scores as outcomes in NMOSD patients after removing the NMOSD outliers

Chemokines	Outcomes	Adjusted β	R^2	P values	Adjusted for
CCL13	Relapse times	0.509	0.494	0.000	Age, Disease duration
	ARR	0.001	0.330	0.991	Disease duration
	EDSS	0.043	0.179	0.756	Age
CCL11	Relapse times	0.023	0.274	0.858	Age, Disease duration
	ARR	-0.099	0.343	0.411	Disease duration
	EDSS	0.013	0.175	0.924	Age
CCL26	Relapse times	-0.059	0.271	0.649	Age, Disease duration
	ARR	0.078	0.332	0.525	Disease duration
	EDSS	-0.082	0.176	0.549	Age

ARR annual relapse rate, *EDSS* expanded disability status scale
