# Supplementary material for: Elevated Plasma Chemokines for Eosinophils in Neuromyelitis Optica Spectrum Disorders during Remission
Source: Front Neurol. 2018 Feb 12;9:44. doi: 10.3389/fneur.2018.00044 (PMC5819570; doi:10.3389/fneur.2018.00044)
Supplement: Supplementary file 2 [file data_sheet_1.PDF]

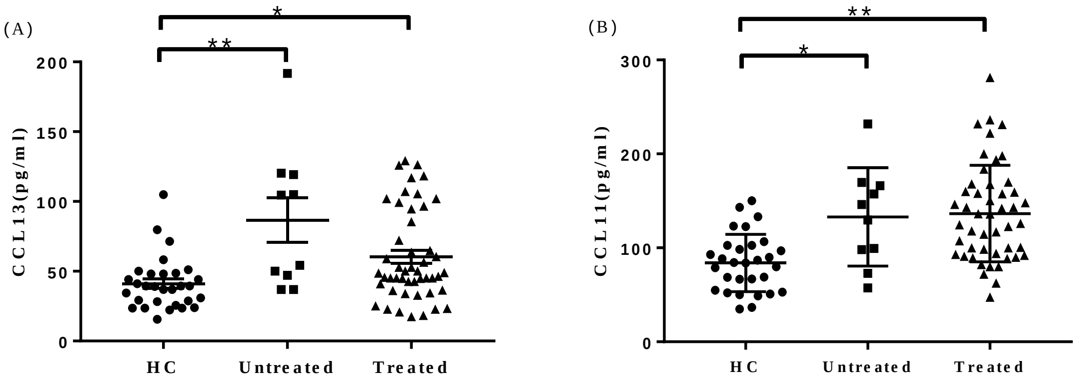

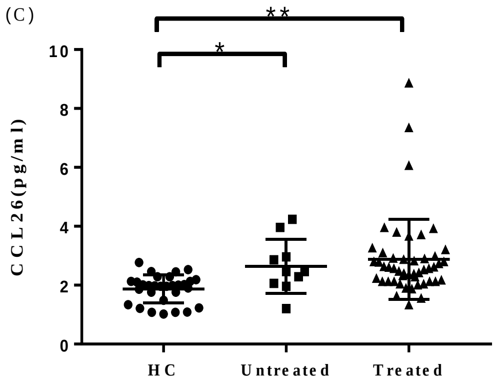

Supplemental Fig.1 Plasma CCL13, CCL11 and CCL26 levels in NMOSD patients untreated and treated with immunosuppressive agents (*n*=48, *n*=18), comparing with HC (mean ± SE). Kruskal-Wallis *H* test and Dunn's post-hoc analysis were used.

$*P < 0.05$, $**P < 0.01$.
