# Supplementary material for: Elevated Plasma Chemokines for Eosinophils in Neuromyelitis Optica Spectrum Disorders during Remission
Source: Front Neurol. 2018 Feb 12;9:44. doi: 10.3389/fneur.2018.00044 (PMC5819570; doi:10.3389/fneur.2018.00044)
Supplement: Supplementary file 3 [file data_sheet_2.PDF]

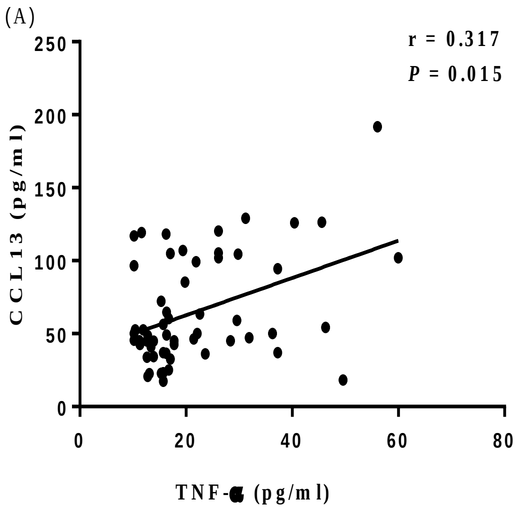

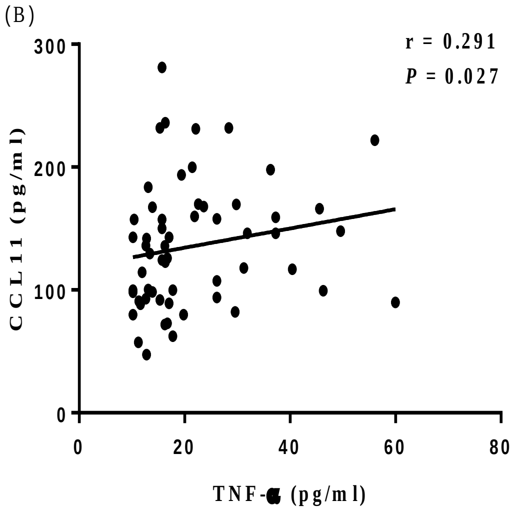

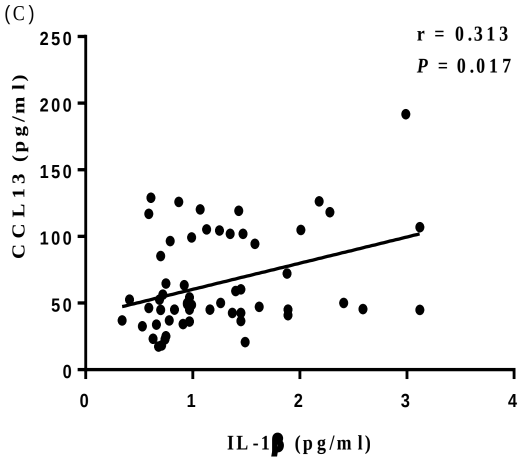

Supplemental Fig.2 Correlation of plasma CCL13, CCL11 with TNF-α and IL-1β levels in NMOSD patients (mean ± SE). Spearman correlation coefficients were used.
