# Supplementary material for: Elevated Plasma Chemokines for Eosinophils in Neuromyelitis Optica Spectrum Disorders during Remission
Source: Front Neurol. 2018 Feb 12;9:44. doi: 10.3389/fneur.2018.00044 (PMC5819570; doi:10.3389/fneur.2018.00044)
Supplement: Supplementary file 4 [file data_sheet_3.PDF]

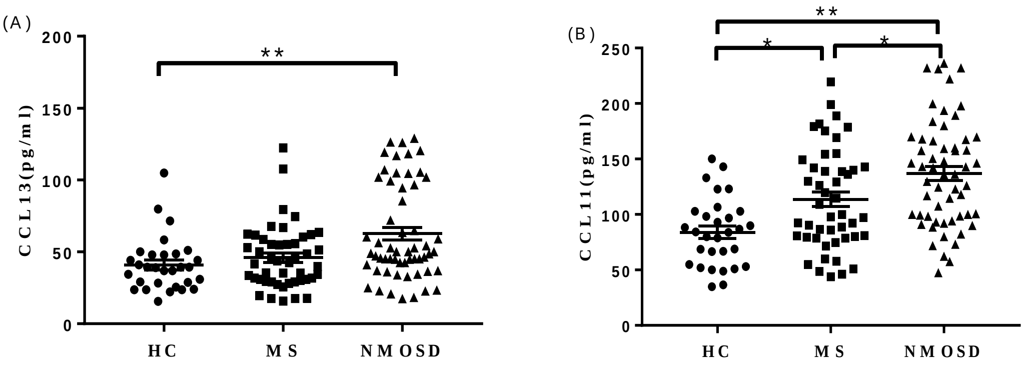

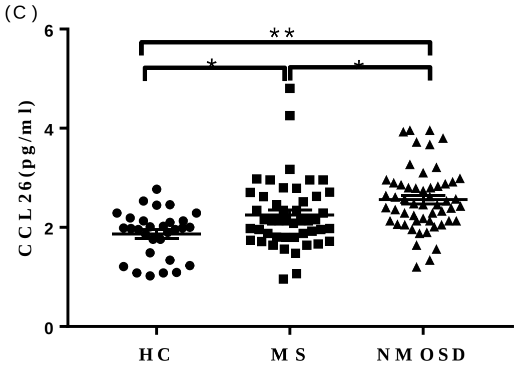

Supplemental Fig.3 Plasma CCL13, CCL11 and CCL26 levels in HC, MS patients and NMOSD patients after removing the NMOSD outliers (mean ± SE). Kruskal-Wallis *H* test and Dunn's post-hoc analysis were used. **P* < 0.05, ***P* < 0.01.
